# ID 326 - Custo-Utilidade dos Cuidados Paliativos Em Oncologia

**DOI:** 10.5327/2237-9622.2025.v34s1.326

**Published:** 2025-11-25

**Authors:** Roberto Carlos Lyra da Silva, Regina Bokehi Nigri, Simone Garruth dos Santos Machado Sampaio, Paulo Alexandre Ribeiro Mora, Sônia Regina de Souza, Antonio Augusto de Freitas Peregrino, Cristiano Bertolossi Marta, Elson Santos de Oliveira, Daniel Aragão Machado

**Keywords:** câncer, cuidados paliativos, análise de custo-utilidade, avaliação econômica em saúde

## Abstract

**Introdução:**

O envelhecimento da população mundial e brasileira tem levado a um aumento significativo na incidência de doenças crônicas não transmissíveis (DCNT), incluindo o câncer. Essa mudança demográfica e epidemiológica destaca a necessidade de cuidados paliativos (CP) eficientes para melhorar a qualidade de vida dos pacientes com doenças avançadas e incuráveis. Objetivos: o estudo avaliou o custo-utilidade dos cuidados paliativos em oncologia, fornecidos por uma unidade do Sistema Único de Saúde (SUS), da rede federal, especializada e de referência em CP, localizada na cidade do Rio de Janeiro.

**Método:**

A pesquisa é uma análise econômica aninhada a uma coorte retrospectiva, utilizando um modelo de Markov para comparar os custos e os resultados dos cuidados paliativos e dos cuidados convencionais para pacientes oncológicos. Os dados foram coletados de quatro unidades assistenciais da mesma instituição de saúde, sendo três de cuidados convencionais e uma de cuidados paliativos. A coorte de pacientes foi selecionada a partir da data de óbito ocorrido durante a internação hospitalar entre os dias 1º a 31 de agosto de 2019. Foram incluídos pacientes maiores de 20 anos, com doença oncológica em estágio avançado com metástase, matriculados na instituição há mais de 60 dias e encaminhados à unidade de cuidados paliativos no mínimo 30 dias antes da data óbito. Os custos foram apurados pela técnica de microcusteio de baixo para cima. A medida de utilidade foi os anos de vida ajustados à qualidade (AVAQ). A utilidade de cada um dos estados de saúde foi extrapolada do estudo de Vanbutsele . (2018). O modelo assumiu que, nas unidades assistenciais HC I, HC II e HC III, os pacientes iniciaram suas jornadas pela emergência ou pelo ambulatório. No cenário alternativo, HC IV, o início se deu na emergência, no ambulatório ou na assistência domiciliar. O estudo foi aprovado pelos Comitês de Ética e Pesquisa (CEPs) da Universidade Federal do Estado do Rio de Janeiro CAAE n.º° 71748523.1.0000.5285, aprovado em 13 de setembro de 2023, e do Instituto Nacional de Câncer CAAE n.º° 71748523.1.3001.5274, aprovado em 24 de outubro de 2023.

**Resultados:**

O custo médio mensal do tratamento de um paciente com câncer em estágio avançado no HC IV foi de R$ 25,72 contra R$ 128,18 do HC I, representando, portanto, um custo 4,98 vezes maior do que o custo no HC IV. Embora a efetividade média mensal possa ter sido 2,33 vezes maior no HC I (0,14 versus 0,06 AVAQ), a relação custo-efetividade nesse hospital foi, na mesma proporção, 2.18 vezes maior do que no HC IV (R$ 899,11 versus R$ 412,06). A razão de custo-efetividade incremental no HC I em relação ao HC IV foi de R$ 1.278,57.

**Conclusão:**

Os cuidados paliativos são essenciais para a gestão eficiente dos recursos em saúde, proporcionando melhor qualidade de vida aos pacientes com câncer avançado. A implementação de políticas que incentivem a adoção de cuidados paliativos desde o diagnóstico é recomendada para otimizar os resultados clínicos e econômicos. Nesse sentido, prestar cuidados paliativos em unidades especializadas pode ser a alternativa mais custo-efetiva para o SUS.

